# Phenotypic and transcriptomic analysis reveals early stress responses in transgenic rice expressing Arabidopsis DREB1a

**DOI:** 10.1002/pld3.456

**Published:** 2022-10-19

**Authors:** Yasmin Vasques Berchembrock, Bhuvan Pathak, Chandan Maurya, Flávia Barbosa Silva Botelho, Vibha Srivastava

**Affiliations:** ^1^ Department of Crop, Soil, and Environmental Sciences University of Arkansas System Division of Agriculture Fayetteville Arkansas USA; ^2^ Present address: Biological and Life Sciences Division, School of Arts and Sciences Ahmedabad University Central Campus Navrangpura Ahmedabad India; ^3^ Department of Agriculture Federal University of Lavras Lavras Minas Gerais Brazil

**Keywords:** abiotic stress, Arabidopsis DREB1a, drought stress, *Oryza sativa*, salinity stress, stress tolerance, transcriptome

## Abstract

Overexpression of Arabidopsis dehydration response element binding 1a (*DREB1a*) is a well‐known approach for developing salinity, cold and/or drought stress tolerance. However, understanding of the genetic mechanisms associated with *DREB1a* expression in rice is generally limited. In this study, *DREB1a*‐associated early responses were investigated in a transgenic rice line harboring cold‐inducible *DREB1a* at a gene stacked locus. Although the function of other genes in the stacked locus was not relevant to stress tolerance, this study demonstrates *DREB1a* can be co‐localized with other genes for multigenic trait enhancement. As expected, the transgenic lines displayed improved tolerance to salinity stress and water withholding as compared with non‐transgenic controls. RNA sequencing and transcriptome analysis showed upregulation of complex transcriptional networks and metabolic reprogramming as *DREB1a* expression led to the upregulation of multiple transcription factor gene families, suppression of photosynthesis, and induction of secondary metabolism. In addition to the detection of previously described mechanisms such as production of protective molecules, potentially novel pathways were also revealed. These include jasmonate, auxin, and ethylene signaling, induction of *JAZ* and *WRKY* regulons, trehalose synthesis, and polyamine catabolism. These genes regulate various stress responses and ensure timely attenuation of the stress signal. Furthermore, genes associated with heat stress response were downregulated in *DREB1a* expressing lines, suggesting antagonism between heat and dehydration stress response pathways. In summary, through a complex transcriptional network, multiple stress signaling pathways are induced by DREB1a that presumably lead to early perception and prompt response toward stress tolerance as well as attenuation of the stress signal to prevent deleterious effects of the runoff response.

## 1 INTRODUCTION

*Arabidopsis thaliana* transcription factor, dehydration response element binding 1a (DREB1a), is induced by cold, drought, and salinity and considered highly promising for engineering abiotic stress tolerance in plants (Kasuga et al., 1999; Smirnoff & Bryant, 1999). Although its constitutive overexpression is deleterious to plant growth, its conditional expression under abiotic stress‐inducible *A. thaliana RD29a* promoter minimally affects plant growth and confers stress tolerance (Ito et al., 2006; Kasuga et al., 1999). Further, because *RD29a* is a stress‐induced gene, it could promote *DREB1a* expression sustainably during stress conditions. *RD29a* promoter strongly induced *DREB1a* expression under salinity, cold, and drought conditions in Arabidopsis (Kasuga et al., 1999), and the expression of *RD29a:DREB1a* conferred tolerance to salinity, drought, and/or freezing stress in diverse plant species including rice (Datta et al., 2012; Ganguly et al., 2020; Geda et al., 2019; Latha et al., 2019; Muthurajan et al., 2021; Oh et al., 2005; Ravikumar et al., 2014), peanuts (Bhalani et al., 2019; Bhatnagar‐Mathur et al., 2007; Devi et al., 2011; Sarkar et al., 2016), chickpea (Anbazhagan et al., 2015), soybean (de Paiva Rolla et al., 2014), chrysanthemum (Hong et al., 2006; Ma et al., 2010), potato (Behnam et al., 2007; Shimazaki et al., 2016), and *Salvia* sp. (Wei et al., 2016). It is now well established that enhanced expression of *DREB1a* by stress‐regulated promoters such as *RD29a* is an effective strategy for conferring abiotic stress tolerance. However, whether a single gene will be sufficient to manage field‐level stress conditions is debatable. It is likely that stress tolerance genes such as *DREB1a* will have to be co‐located with other genes that either confer stress tolerance or help overcome growth or yield trade‐offs imposed by stress response genes (Kudo et al., 2019; Shailani et al., 2021).

In the present study, transgenic rice developed through Cre‐*lox* recombination‐mediated site‐specific integration of multigene cassette were used (Srivastava & Ow, 2002). The rice lines contained *RD29a:DREB1a* gene along with four marker genes and the Cre‐*lox* method facilitated precise full‐length integration of the multigene cassette into a predetermined genomic locus (Pathak & Srivastava, 2020). Thus, the functionality of *DREB1a* in *japonica* rice was tested from a stacked locus consisting of five genes: neomycin phosphotransferase II (*NPT II*), β‐glucuronidase (*GUS*), green fluorescence protein (*GFP*), red fluorescence protein (ppor*RFP*), and *DREB1a*.

Under the stress condition, Arabidopsis DREB1a interacts with *cis*‐acting *DRE* (dehydration responsive elements: RCCGAC, where R = A/G) for the regulation of stress responsive genes (Dubouzet et al., 2003; Yamaguchi‐Shinozaki & Shinozaki, 1994). In Arabidopsis, its overexpression induces various stress‐responsive genes that contain *DRE* in response to drought, salinity, and cold stress (Kasuga et al., 1999; Maruyama et al., 2004). However, in spite of its popularity in crop biotechnology, only a limited investigation has been done to reveal the downstream targets of DREB1a in crop species such as rice. Microarray analysis of *japonica* rice seedlings expressing *ZmUbi1:DREB1a* (strong constitutive expression) found potential DREB1a targets but did not shed light on the mechanisms associated with drought/salinity tolerance (Oh et al., 2005). Ito et al. (2006) found proline and various sugar accumulations in rice overexpressing *DREB1a* or its rice ortholog (*OsDREB1a*) and proposed accumulation of these molecules as part of the stress tolerance mechanism. Latha et al. (2019) using microarray showed induction of *DRE*‐containing genes encoding chloroplast structure and function in *RD29a:DREB1a indica* rice exposed to drought at reproductive stage. Similarly, RNA‐seq analysis of *Salvia* sp. expressing *RD29a:DREB1a* showed induction of genes involved in photosynthesis, signal transduction, and transcriptional activation, as well as carbohydrate transport, metabolism, and protein protection (Wei et al., 2016). Finally, a study on *DREB1a* overexpressing chrysanthemum found similarities between DREB1a regulon of chrysanthemum and that of rice and Arabidopsis. This study used suppressive subtractive hybridization approach for identifying DREB1a targets in chrysanthemum (Ma et al., 2010).

Here, we analyzed transgenic rice lines (cv. Taipei‐309) that contained a stack of five genes, including *RD29a:DREB1a*. Taipei‐309 is a chilling‐tolerant but drought‐ and salinity‐sensitive *japonica* rice (Li et al., 2010). Therefore, only drought and salinity stress tolerance were studied at the young vegetative stages in the artificial media or greenhouse. The transgenic lines expressing *RD29a:DREB1a* showed significantly improved tolerance to both salinity (100–150 mM NaCl) and drought (10% soil water content [SWC]) in comparison with the non‐transgenic controls. Transcriptomic analysis showed that induction of *DREB1a* led to the onset of complex transcriptional reprogramming along with hormone signaling and accumulation of a variety of protective molecules that protect the cellular components from the damaging effects of the stress. As some of these mechanisms have been reported in drought/cold/salinity stressed plants (Yamaguchi‐Shinozaki & Shinozaki, 2006; Zhang et al., 2021), their enrichment in *DREB1a* overexpressing lines indicates involvement of multiple pathways and cross‐talks among various transcription factors during early phase of the exposure to adverse condition toward preparing the plant to survive the dehydration stress.

## 2 MATERIALS AND METHODS

### 2.1 Plant materials

The previously described multigene stacked lines of rice cv. Taipei‐309 were used in the present study for drought and salinity stress (Pathak & Srivastava, 2020). These lines contained *RD29a:DREB1a* gene in addition to four marker genes each expressed by strong constitutive or inducible promoters (*NPTII*, *GUS*, *GFP*, and *pporRFP*). These lines were developed using a multigene vector, pNS64, in the T5 founder line using Cre‐*lox* mediated site‐specific integration approach (Srivastava & Ow, 2002). Because each multigene stacked line is isogenic, and harbored identical transgene integration and genomic location pattern, T1 seeds from homozygous lines carrying biallelic integration of pNS64 construct (lines #11 and #29) or the hemizygous lines containing monoallelic integration (lines #9 and #10) were used in the study (see Pathak & Srivastava, 2020). Here, phenotypic data from homozygous lines are presented. For simplicity, the multigene transgenic lines harboring *RD29a:DREB1a* are referred to as “transgenic” (T) and the parental T5 line as “non‐transgenic” (N) in this study.

### 2.2 Salinity stress analysis

Rice seeds were germinated on MS½ medium solidified with 2% Phytagel, and the seedlings at S3 stage, that is, having a prophyll emerged from coleoptile, were transferred to borosilicate glass tubes containing MS½ medium supplemented with 0 (control), 100, or 150 mM NaCl. The seedlings were kept under light intensity of 20–40 μE/m^2^/s at 28°C until third leaf stage (V3) (Moldenhauer et al., 2018). The experiment was conducted in complete randomized design in a factorial scheme (2 × 3), two genotypes in three salinity conditions, with 15 replications and a single plant as the experimental unit. The number of chlorotic and necrotic leaves was evaluated daily, and the growth rate (GR) of the seedlings was evaluated every 3 days using the formula $\mathrm{GR}=l_{x}-l_{y}$, in which $l_{x}$ is the length of the seedling (cm) measured from the base of the stem to the end of the extended leaves on the day and $l_{y}$ is the same length (cm) observed in the previous measurement.

### 2.3 Water withholding stress analysis

Rice seeds were germinated on MS½ medium until the S3 stage when it was transferred individually to pots of 0.6 L containing 45 g of a dry mixture of sphagnum peat moss and perlite (9:1), PRO‐MIX LP15®. Before sowing, each pot was weighed, saturated with water, and placed in a tray filled with water for 2 days. The pots were then removed from the tray and placed on a grid until the water drained. When no dripping was observed, the pots were weighed again, and this weight was considered as the field capacity (100% SWC). The weight of SWC at the field capacity was calculated as the difference between the pot weight with water‐saturated soil and with dry soil (Almeida et al., 2016). All the plants were kept at field capacity for 2 weeks, when half of the plants were subjected to water stress. The irrigation in these plants was suspended, and the pots were weighed every day until they reached 10% of SWC (representing a severe stress). Subsequently, the plants were irrigated again for 1 week for recovery. The plants were kept in growth chamber at 28°C and 300–500 μE/m^2^/s light intensity with a 12‐h photoperiod during throughout the experiment. A completely randomized design in a factorial scheme (2 × 2) with 10 replications was used with one plant as the experimental unit. The two treatments consisted of two water regimes, well‐watered control (100% SWC) and water stressed condition (10% SWC). The plants in stress were checked every day to determine the SWC at which the plants started to display the first symptoms of withering, chlorosis, and leaf senescence. After 1 week of recovery, all the plants were analyzed for percentage of leaves with chlorosis and percentage of leaves with necrosis.

### 2.4 Statistical analysis

The phenotypic data were statistically analyzed by analysis of variance (ANOVA) and the Tukey test for comparisons among treatment; the means was applied with 5% as the level of significance using PROC MIXED of SAS (SAS, 2013) and R (R Core Team, 2017) software using fBasics and agricolae packages.

### 2.5 RNA‐seq

Seeds of the transgenic (T) and non‐transgenic controls (N) were germinated in Petri dishes containing MS½ media and grown in 16/8 h (day/night) condition at 28°C in 150 μE/m^2^/s light intensity. Ten‐day‐old seedlings were divided into two groups: cold‐shock treatment (CS) and the control treatment at the room temperature (RT). Cold‐shock treatment was done by placing the plates on crushed ice in 4°C chamber for 20 h starting at 2 p.m. and ending at 10 a.m. on the next day. The control plates were kept at ambient RT during the same time. At the end, the aerial portion of seedlings was separated from the roots and snap‐frozen for total RNA isolation using Trizol reagent (Thermo Fisher Scientific, USA). RNA was treated with DNase I, and its concentration and quality were measured with Qubit 2.0 fluorometer (Thermo Fisher Scientific, USA). Subsequently, RNA samples of all six seedlings (biological replicates) were pooled for each genotype and treatment, yielding four pooled groups: T_CS, T_RT, N_CS, and N_RT. Two technical replicates of each pooled group were submitted for cDNA library synthesis, and Illumina sequencing was done at Novogene Inc. RNA quality and quantity was determined by High Sensitivity RNA TapeStation D1000 ScreenTape (Agilent Technologies Inc., California, USA) and Qubit 2.0 RNA High Sensitivity assay (Thermo Fisher Scientific, USA), respectively. Paramagnetic beads coupled with oligo d(T)25 were combined with total RNA to isolate poly(A)+ transcripts based on NEBNext® Poly(A) mRNA Magnetic Isolation Module manual (New England BioLabs Inc., Massachusetts, USA). All libraries were constructed (average insert size ~270 bp) according to the NEBNext® Ultra™ II Directional RNA Library Prep Kit for Illumina® (New England BioLabs Inc., Massachusetts, USA) and sequenced with Illumina® 8‐nt dual‐indices for paired‐end sequencing on Illumina® HiSeq (Illumina, California, USA) with a read length configuration of 150 paired‐end for 40 million paired‐end reads per sample (20 million in each direction). The data discussed in this publication have been deposited in NCBI's Gene Expression Omnibus (Edgar et al., 2002) and are accessible through GEO Series accession number GSE185088 (https://www.ncbi.nlm.nih.gov/geo/query/acc.cgi?acc=GSE185088).

### 2.6 Differential gene expression analysis

The differential gene expression analysis was performed using main GALAXY platform (usegalaxy.org) version 2.7.5b (Afgan et al., 2018). The raw data quality in the fastq format was assessed using FastQC tool. Further quality check and removal of adapter sequences were done by TRIMMOMATIC tool using SLIDINGWINDOW, MINLEN, and AVGQUAL parameters. The raw reads were mapped on RNA‐STAR using rice genome (*O. sativa* japonica group) downloaded from Ensemble version 48 in the FASTA format (ftp://ftp.ensemblgenomes.org/pub/plants/release‐48/fasta/oryza_sativa/dna/Oryza_sativa.IRGSP‐1.0.dna.toplevel.fa.gz). The Ensemble version 48 cDNA annotation in GTF format (ftp://ftp.ensemblgenomes.org/pub/plants/release-49/gtf/oryza_sativa/Oryza_sativa.IRGSP-1.0.48.gtf.gz) was used for generating the genome indexing. The normalized counts were obtained from Featurecounts version 1.6.4 (Liao et al., 2014) using Gene‐ID “t” read count, gene_id features, and differentially expressed genes (DEG) were obtained using edgeR with log_2_ fold change (FC) ≥ 2, *P*‐value ≤ .05, using *P*‐value adjustment method of Benjamin and Hochberg (Liu et al., 2015). The genes were considered differentially expressed if values were above the threshold (log_2_FC ≥ 2 or ≤−1, FDR ≤ 0.01) in the comparisons of cold‐stressed *RD29a:DREB1a* transgenic lines (T_CS) versus cold‐stressed non‐transgenic control (N_CS) and in room‐temperature *RD29a:DREB1a* transgenic lines (T_RT) versus room‐temperature non‐transgenic controls (N_RT). To determine the read counts for transgenes stacked in the transgenic line, the reads from T_CS and T_RT were filtered using BAM Split tool into mapped and unmapped reads, and unmapped reads were mapped against *NPTII* (U55762.1), *GFP* (U55762.1), *GUS* (AF485783), *DREB1a* (NM_118680.2), and *pporRFP* (DQ206380.1) sequences downloaded from NCBI in the fasta format using “map with BWA version” 0.07.17.4 (Li & Durbin, 2010) containing “IS” algorithm mode. These aligned reads were sorted using Samtools, and reads were obtained using Samtools idxstat. Heatmaps of DEGs, k‐means clusters, and gene network were developed using iDEP.94 (http://bioinformatics.sdstate.edu/idep94/; Ge et al., 2018). Gene Ontology (GO) and the gene set enrichment analysis for the DEGs were also carried out on iDEP.94, and Kyoto Encyclopedia of Genes and Genomes (KEGG) analysis was performed on BLASTKOALA (https://www.kegg.jp/blastkoala/; Kanehisa et al., 2016).

### 2.7 Search of DRE elements

Drought response element (DRE) in DEGs was searched using PLANT PAN 3.0 (Support ≥ 70% frequency of promoters containing the transcription factor [TF]; Chow et al., 2019). For this purpose, 2‐kb sequence upstream of the start codon of 683 upregulated genes (log_2_ FC ≥ 2, FDR < 0.01) were obtained from BioMart in Plant Ensemble v48. The extracted regions of these genes were searched for DRE core‐motif (A/GCCGAC) #292.

### 2.8 qRT‐PCR

The pooled RNA for each genotype and treatment was used for qRT‐PCR. One microgram DNase‐treated RNA was converted to cDNA using Prime RT‐PCR kit (Takara Bio Inc.). The qPCR was performed on 11 selected DEGs containing DRE elements. The primers for each gene are listed in Table S1 with *Os7Ubiquitin* serving as the reference gene. All reactions were performed in two technical replicates, and fold change in transgenic lines relative to non‐transgenic line under the same treatment (RT or CS) was calculated by 2^ddCT method (Livak & Schmittgen, 2001).

## 3 RESULTS

### 3.1 Seedling response to salinity stress

**FIGURE 1:**
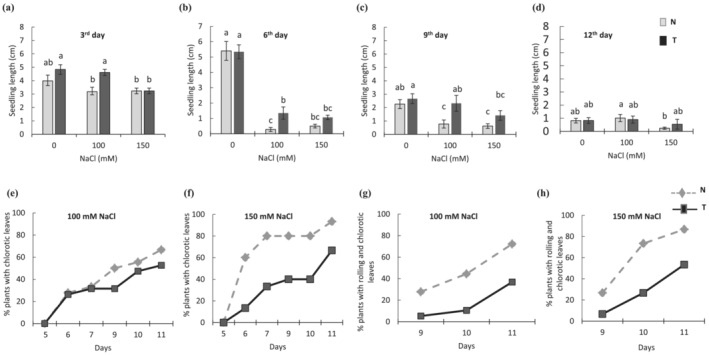
Salinity stress assay. Three‐day‐old seedlings of *RD29a:DREB1a* transgenic lines (T) and non‐transgenic (N) controls grown in MS½ media containing 0, 100, or 150 mM NaCl. (a–d) Seedling growth rates (increase in the length of the seedling in centimeters) recorded on 3rd, 6th, 9th, and 12th day of the treatment. (e,f) Percentage of plants with chlorotic leaves under salinity stress of 100 or 150 mM NaCl. (g,h) Percentage of plants with rolling and chlorotic leaves under salinity stress of 100 or 150 mM NaCl. Different letters indicate differences by Tukey test (*n* = 15). The error bars indicate standard errors. N and T genotypes are shown as gray and black bars, respectively, in (a–d) and as dashed and solid lines in (e–h).

*RD29a:DREB1a* transgenic (T) lines and non‐transgenic (N) controls were tested for the salt stress assay with two different concentrations of NaCl. Significant genotype effect (*P* < .05) was observed for all evaluated traits, indicating a difference between T and N lines. At the beginning of vegetative stage (third day), T seedlings showed a slightly higher growth rate (increase in seedling length) under non‐stress condition and greater tolerance to 100 mM NaCl (Figure 1a). The N seedlings had a negative response to 100 mM salt stress, decreasing 20.2% of growth rate when compared with the salt control (0 mM). The maximum reduction in growth rate in stressed seedlings was observed on the sixth day for both T and N lines (Figure 1b). In 100 mM NaCl, there was a reduction of 4‐fold and 19‐fold, respectively, for T and N in comparison with non‐stress control. However, no significant difference was found in growth rate of T and N seedlings under more severe stress (150 mM NaCl). On the ninth day, the growth rate for T plants was again greater than N plants for both salinity stress conditions (Figure 1c). Although T seedlings had a small reduction in growth rate under 100 mM NaCl, their performance was generally similar to that of N seedlings under no stress. The N lines, on the other hand, were more sensitive to salinity showing a reduction of 2.9‐fold under 100 mM and 3.6‐fold under 150 mM NaCl. On the 12th day, growth rate slowed down; however, performance of T plants under salt stress was similar to those under non‐stress control, whereas N plants showed a significant reduction in growth in 150 mM NaCl (Figure 1d).

Until fifth day, no leaf chlorosis was observed in plants under stress or the control condition (Figure 1e,f). By the seventh day on 100 mM NaCl, both genotypes had 30% of seedlings with chlorotic leaves. However, this percentage increased more sharply over the days in N lines, showing, at the end, 14% more plants with chlorosis than T lines (Figure 1e). In 150 mM NaCl, this difference was greater and could be observed starting the sixth day, reaching in 93% and 67% in N and T seedlings, respectively (Figure 1f). The percentage of chlorotic leaves that also showed rolling, a symptom of severe stress, was greater in N plants with a difference of 35% (100 mM NaCl) and 34% (150 mM NaCl) compared with T plants (Figure 1g,h **)**. Overall, T seedlings displayed higher growth rate and reduced leaf necrosis as compared with N seedlings after 12–15 days of continuous stress (Figure S1). However, because salt stress imposes both ionic and osmotic pressures in rice (Castillo et al., 2007), the observed growth reduction and leaf chlorosis could be caused by osmotic stress.

### 3.2 Plants response to drought stress

**FIGURE 2:**
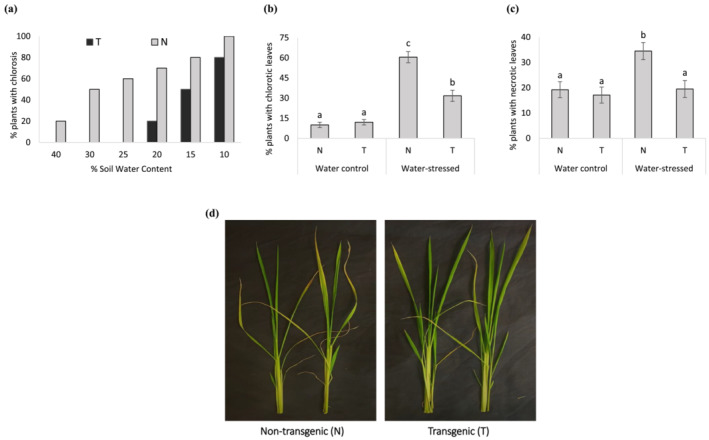
Water withholding assay. Two‐week‐old plants of *RD29a:DREB1a* transgenic lines (T) and non‐transgenic (N) controls maintained at 100% soil water content (SWC) subjected to water withholding until 10% SWC. (a) Percentage of plants with chlorotic leaves under different levels of SWC. (b,c) Percentage of plants with chlorotic and necrotic leaves at the end of the experiment in water control (100% SWC) and water stressed (10% SWC) plants. (d) Shoots of N and T plants after 7 days of water recovery. Note greater chlorosis and leaf wilting (wavy leaves) in the N shoots as compared with T shoots. Different letters indicate differences by Tukey test (*n* = 10). The error bars indicate standard errors.

After submitting rice plants to drought stress, the phenotypic symptoms caused by stress were evaluated in the vegetative stage. After withholding water, N plants started to show the first symptoms of stress in leaves (chlorosis) at 40% of SWC, although no symptoms were observed in T plants (Figure 2a). At 20% SWC, 70% of N plants exhibited the leaf chlorosis as the symptom of stress, although T lines had just started to develop first symptoms of stress. The *DREB1A* gene was associated with increased tolerance to drought as majority of T plants were asymptomatic in 20% SWC. Further, in maximum stress (10% SWC), a few T plants looked normal without any apparent symptoms, whereas all N plants showed chlorosis (Figure 2a). At the end of the experiment (10% SWC), lower number of T plants showed chlorotic and necrotic leaves (Figure 2b,c) and overall looked healthier than N plants (Figure S2). Finally, N plants had more wilted and chlorotic leaves even after the water recovery time than T plants (Figure 2d).

### 3.3 Transcriptome analysis of cold‐stress‐induced DREB1a transgenic lines

**FIGURE 3:**
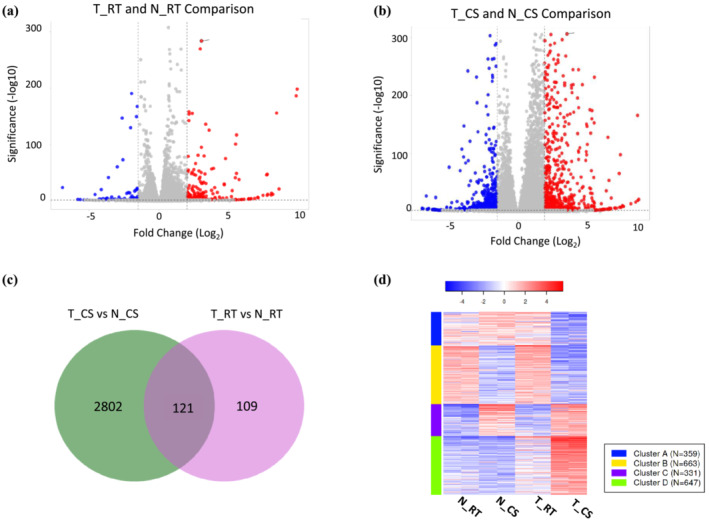
Differentially expressed genes (DEGs) in *RD29a:DREB1a* transgenic lines upon 20 h cold‐shock treatment (T_CS) or under room temperature control condition (T_RT) in comparison with non‐transgenic controls in the respective treatment (N_CS or N_RT). (a,b) Volcano plot showing DEGs between two genotypes upon RT or CS treatments. Blue and red represent downregulated and upregulated genes, respectively, and dashed lines show log_2_ cut off for differential expression; (c) Venn diagram showing distribution of DEGs in T_CS and T_RT in comparison to N_CS and N_RT, respectively (Total number of genes = 31,468; FDR cutoff 0.01, log_2_FC ≥ 2 or ≤−1); (d) k‐means clustering of top 2000 most variable genes using normalized expression counts. Heatmap shows lower (blue) to higher (red) expression of the cluster of genes in N_RT, N_CS, T_RT, and T_CS including two replicates of each genotype_treatment. Data analyzed on iDEP.94 http://bioinformatics.sdstate.edu/idep94/ (Ge et al., 2018).

To understand the potential mechanism by which DREB1a provides stress tolerance to rice, RNA‐seq was done on cold‐stressed (CS) and room temperature controls (RT) of the two genotypes: transgenic line expressing *RD29a:DREB1a* (T) and non‐transgenic controls (N) (Table S2). The DEGs were identified through comparisons between genotypes (T vs. N) in the respective treatment (CS vs RT) (Figure 3a,b). *RD29a* promoter is induced by multiple abiotic stresses including cold stress (Msanne et al., 2011). Earlier, transgenic rice lines #9, #11, and #29 were characterized to strongly induce *DREB1a* upon cold treatment (Pathak & Srivastava, 2020). Consistent with that, RNA‐seq comparison of cold‐stressed transgenic lines with the room temperature control (T_CS vs. T_RT) showed ~49‐fold higher read counts of *DREB1a*, although no significant change (<2‐fold) was observed in other transgenes (marker genes) stacked in the same locus (Table S3).

A comparison of T_CS versus N_CS and T_RT versus N_RT showed 2802 unique DEGs in T_CS, 109 unique DEGs in T_RT, and 121 DEGs common to T_CS and T_RT (Figure 3c). The k‐means clustering of top 2000 most variable genes using normalized expression values showed that T_CS contained a large cluster of strongly upregulated genes and a smaller cluster of downregulated genes, and the two genotypes at room temperature (T_RT and N_RT) showed a similar expression pattern (Figure 3d). These observations fit the hypothesis that upon induction of *RD29a:DREB1a* by cold‐shock (CS) several genes are induced through binding of DREB1a to the promoters of the target genes, and the leaky expression of *RD29a* promoter at RT results in the induction of a smaller set of the DREB1a targets at RT. The list of DEGs were further filtered with the criteria of *P*‐value < .01 and log2FC ≥ 2. The resulting 2069 genes were used for the GO and pathway analysis.

### 3.4 Functional annotation of genes induced in *RD29a:DREB1a* transgenic line upon cold stress

**FIGURE 4:**
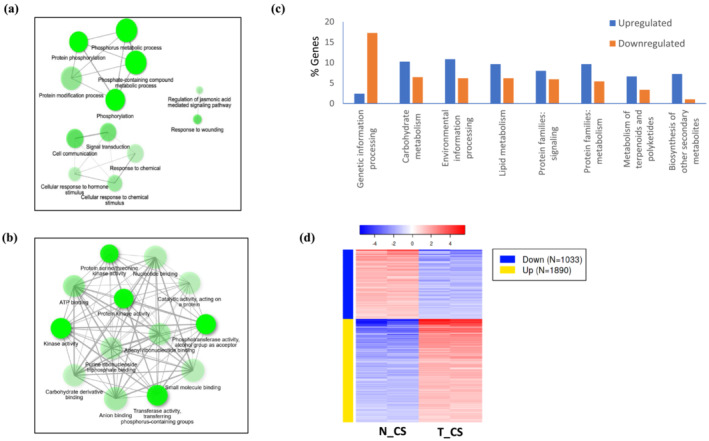
Rice genetic pathways regulated by DREB1a. Network of (a) biological processes and (b) molecular functions associated with upregulated genes in *RD29a:DREB1a* transgenic line upon cold‐shock in comparison to cold‐shocked non‐transgenic controls. Darker nodes are more significantly enriched gene sets. Bigger nodes represent larger gene sets. Thicker edges represent more overlapped genes. Data analyzed on iDEP.94 http://bioinformatics.sdstate.edu/idep94/ (Ge et al., 2018). (c) Upregulated or downregulated Kyoto Encyclopedia of Genes and Genomes (KEGG) pathways in cold‐shocked transgenic line expressing *RD29a:DREB1a* in comparison to cold‐shocked non‐transgenic controls. (d) Cluster of upregulated and downregulated genes in two technical replicates of transgenic (T) or non‐transgenic (N) lines treated with cold‐shock (CS) (FDR < 0.01, log_2_FC ≥ 2 or ≤−1).

The enriched GO biological processes (BP) in T_CS included protein phosphorylation, signal transduction, response to chemical and hormone stimulus, and jasmonic acid (JA)‐mediated signaling, GO molecular functions (MF) included kinase activity and protein Ser/Thr kinase activity, nucleotide binding, and GO cellular component (CC) included plasma membrane and cell periphery (Figures 4a,b and S3a). The downregulated genes in T_CS, on the other hand, were associated with response to heat, protein folding, cell cycle process, and photosynthesis, light harvesting in photosystem I (Figure S3b). The gene set enrichment analysis showed that upregulated DEGs in T_CS were involved in protein serine/threonine kinase activity, kinase activity, catalytic activity, protein kinase activity, oxidoreductase activity, and DNA binding transcription factor activity. The downregulated genes in T_CS were involved in unfolded protein binding, microtubule binding, tubulin binding, protein heterodimerization activity, and microtubule motor activity. Among the biological pathways, plant hormone signal transduction and α‐linolenic acid metabolism were highly enriched in the upregulated genes, and diterpenoid biosynthesis, DNA replication, photosynthesis‐antennae proteins, and protein processing in endoplasmic reticulum pathways were enriched in downregulated genes. In T_RT, the upregulated genes were enriched in DNA‐binding transcription factor activity and transcription‐related processes, although no pathways were significantly expressed. In the downregulated genes, no significant pathways were detected; however, two genes involved in cutin, suberin, and wax biosynthesis were enriched.

The KEGG pathways were analyzed only for T_CS. This analysis showed that a higher percentage of downregulated genes were associated with genetic information processing, whereas a higher percentage of upregulated genes were associated with carbohydrate metabolism, environmental information processing, lipid metabolism, signaling, and secondary metabolite synthesis, among others (Figure 4c). Corroborating with the GSEA, the upregulated genes were associated with phenylpropanoid biosynthesis, flavonoid biosynthesis, α‐linolenic acid metabolism, and amino acid metabolism, whereas MAPK signaling, hormone signaling, and calcium signaling were among the upregulated environmental information processing pathways. In summary, GSEA and KEGG pathway analysis indicate induction of TF genes that potentially regulate secondary metabolites and sugar metabolism.

**FIGURE 5:**
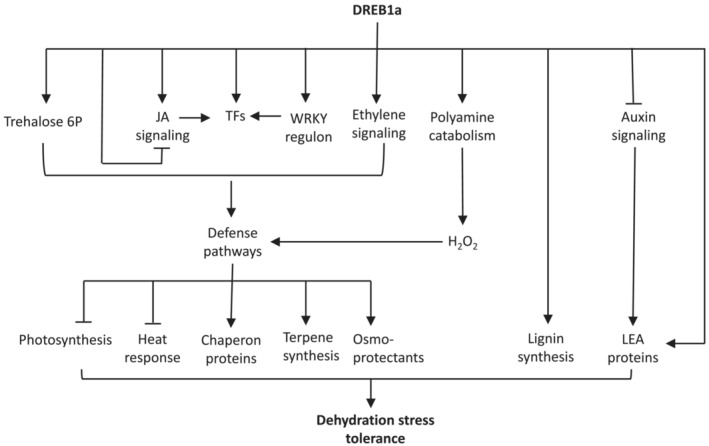
Conceptual map of biochemical and signaling pathways regulated by DREB1a. Sustained induction of DREB1a leads to the expression of transcription factor (TF) genes that positively regulate defense pathways. Production of signal molecules such as trehalose‐6‐P and hydrogen peroxide (H_2_O_2_) through induction of genes encoding metabolic enzymes reinforce defense pathways. Through direct or indirect interactions, DREB1a induces lignin biosynthesis, production of osmoprotectants, secondary metabolites, and chaperon proteins that fortify cell wall, scavenge oxidants, and protect cellular membranes. To support stress response, resources are diverted from primary metabolism through suppression of photosynthesis and antagonistic pathways such as heat stress response, eventually leading to stress tolerance. Upregulations and suppressions of pathway/component are shown by arrows and stubbed lines, respectively.

The mechanisms by which DREB1a confers stress tolerance in rice was further investigated by analyzing the cluster of DEGs based on K‐means clustering (FDR < 0.01, log_2_FC ≥ 2) and the function of the top DEGs in T_CS versus N_CS comparison (Figure 4d). The two approaches converged to mostly common outcomes that shed light on likely pathways involved in the process (Figure 5). The most significant pathways are described below.

### 3.5 JA signaling

The GO analysis of the cluster of DEGs uniquely upregulated in T_CS indicated a role for JA signaling and genes involved in response to wounding, among others (Figure 4a and Table 1). The top JA signaling genes in T_CS include JAZ (JASMONATE ZIM‐DOMAIN) family of genes: *OsJAZ1*, *OsJAZ2*, *OsJAZ5*, *OsJAZ8/9*, and *OsJAZ11* (Table 2). Proteasome‐mediated degradation of JAZ is a key step in unleashing JA signaling (Chini et al., 2007; Thines et al., 2007). Of the five upregulated *JAZ*, *OsJAZ5* and *OsJAZ7* contained three *DRE* elements each. On the other hand, genes associated with α‐linolenic acid metabolism, including lipoxygenases (*LOX1*) and 12‐oxophytodienoic acid reductase (*OPR*) with putative functions in JA biosynthesis, are also upregulated in T_CS. Some of *LOX1* genes and *OPR1* contain *DRE* elements (Table 2).

**TABLE 1:** GO terms associated with upregulated genes in cold‐stressed *RD29a:DREB1a* transgenic line (T_CS) relative to the cold‐stressed non‐transgenic line (N_CS)

No. of genes	GO: Biological processes^a^
243	Phosphorus metabolic process
164	Protein phosphorylation
242	Phosphate‐containing compound metabolic process
188	Phosphorylation
129	Cell communication
22	Response to wounding
114	Signal transduction
240	Protein modification process
87	Cellular response to chemical stimulus
13	Regulation of jasmonic acid‐mediated signaling pathway
56	Cellular response to hormone stimulus
119	Response to chemical
No. of genes	GO: Molecular functions^a^
188	Kinase activity
175	Phosphotransferase activity, alcohol group as acceptor
160	Protein kinase activity
133	Protein serine/threonine kinase activity
262	Nucleotide binding
244	Carbohydrate derivative binding
No. of genes	GO: Cellular component^a^
190	Plasma membrane
215	Cell periphery
33	Intrinsic component of plasma membrane

^a^ GO terms associated with differentially expressed genes (FDR < 0.01, log2 fold change ≥ 2).

**TABLE 2:** Biological pathways induced by DREB1a in rice

Pathway/function	Genes	Gene ID	Log2 FC^a^	No. of DRE^b^
Jasmonic acid signaling (*JAZ* regulon)	OsJAZ1/TIFY11d	Os10g0392400	3.6	‐
	OsJAZ2/TIFY11c‐like	Os03g0180900	3.7	‐
	OsJAZ5/TIFY10a	Os03g0402800	3.0	1
	OsJAZ8/9/TIFY10c	Os09g0439200	2.5	3
	OsJAZ11/TIFY9	Os04g0395800	5.4	3
α‐Linolenic acid metabolism (JA synthesis)	OsLOX1a	Os03g0738600	3.0	‐
	OsLOX1b	Os03g0700700	2.4	2
	OsLOX2	Os08g0508800	2.4	‐
	OsOPR1	Os06g0216300	5.1	1
	OsOPR6	Os06g0215500	7.6	‐
Auxin signaling	OsGH3‐6	Os05g0143800	2.3	‐
	OsGH3‐8	Os07g0592600	2.1	5
*WRKY* genes	OsWRKY8	Os05g0583000	6.7	1
	OsWRKY108	Os01g0821300	5.5	1
	OsWRKY24	Os01g0714800	5.2	1
	OsWRKY76	Os09g0417600	4.5	1
	OsWRKY67	Os11g0117400	3.6	‐
	OsWRKY113	Os06g0158100	2.5	‐
	OsWRKY62	Os09g0417800	2.2	‐
	OsWRKY15	Os01g0656400	2.0	1
Ethylene signaling (*ERF* genes)	OsERF67	Os07g0674800	7.8	2
	OsERF118	Os11g0168500	7.6	2
	OsERF27	Os02g0676800	5.1	‐
	OsERF109a	Os02g0764700	4.6	2
	OsERF108	Os02g0781300	4.2	‐
	OsERF109b	Os08g0474000	2.5	1
Other transcription factors	ONAC59	Os01g0862800	6.1	2
	bHLH148‐like	Os01g0773800	8.9	‐
Dehydrin	OsLEA22	Os01g0702500	9.0	‐
	OsLEA2	Os01g0314800	2.0	2
Universal stress protein	OsUSP36	Os10g0437500	5.2	1
Chaperon proteins	Chaperon protein dnaJ	Os01g0702450	4.2	‐
		Os08g0452900	3.6	1
Early response dehyd.	OsERD7, chloroplastic	Os03g0241900	3.0	3
Trehalose biosynthesis	Trehalose‐6‐phosphate	Os08g0445700	3.6	1
	Synthase	Os08g0414700	2.3	‐
Lignin biosynthesis	OsCCR	Os08g0277200	6.2	1
	OsC4H	Os02g0467000	6.1	1
Terpene synthesis	OsGGPS7	Os01g0248701	4.8	‐
	OsTPS3	Os02g0121700	5.4	‐
	OsTPS23	Os04g0344400	6.3	‐
Polyamine catabolism	OsPOA4	Os04g0671200	3.7	3
	OsPOA6	Os09g0368200	4.6	‐

^a^ Fold change (Log_2_FC) in cold‐treated transgenic line (T_CS) compared with the cold‐treated non‐transgenic controls (N_CS).

^b^ Number of DRE core motif (A/GCCGAC) in 2‐kb sequence upstream of the start site.

### 3.6 Auxin signaling

Cellular response to hormone stimulus was among the upregulated biological processes in T_CS (Table 1). Most notably, *GH3‐6* and *GH3‐8* that encode indole‐3‐acetic acid (IAA)‐amido synthetases are upregulated in T_CS (Table 2). These enzymes catalyze conjugation of IAA with amino acids leading to its depletion (Staswick et al., 2005). Consistent with our observation, previous studies showed *GH3* induction during cold and drought (Du et al., 2012; Han et al., 2020). The promoter of *OsGH3‐6* lacks *DRE* elements, but that of *OsGH3‐8* contains five *DRE* elements (Table 2). Thus, *OsGH3‐8* could be a direct target of DREB1a. Zhang et al. (2009) showed that induction of *GH3* not only attenuates IAA signaling but also induces expression of late embryogenesis abundant (*LEA*) genes in rice. Many *LEA* genes are induced during desiccation stress; accordingly, *OsLEA22* was ~9‐fold upregulated in T_CS (Table 2). *OsLEA22* gene promoter lacks *DRE* elements; therefore, its upregulation could be mediated through modulation of IAA signaling.

### 3.7 *WRKY* transcription factors

Rice contains a large family of *WRKY* genes, majority of which is uncharacterized so far. Initially, *WRKY* were described to mediate insect and pathogen response, but their role in abiotic stress is becoming increasingly evident (Rushton et al., 2010; Viana et al., 2018). Eight *WRKY* TFs were upregulated in T_CS, five of which contained one *DRE* each (Table 2). Of these, *OsWRKY76*, a transcriptional repressor, has been found to have a direct role in cold tolerance (Yokotani et al., 2013). In addition, *OsWRKY‐8*, *OsWRKY‐24*, and *OsWRKY‐108* are also upregulated >4‐fold in T_CS and are likely involved in DREB1a‐mediated abiotic stress tolerance.

### 3.8 Other transcription factors

Besides *WRKY*, other TFs were also upregulated in T_CS. These include ethylene responsive factors (*ERFs*), basic helix loop helix (*bHLH*), and *NAC* (NAM ATAF1/2 CUC2) (Table 2). Of these, *ERFs* and *NAC* are well‐known positive regulators of abiotic stress tolerance including cold stress (Hoang et al., 2017). Thus, multiple TFs that regulate abiotic stress are significantly upregulated in T_CS, suggesting that DREB1a regulates a complex network of genes associated with stress tolerance.

### 3.9 Protective molecules

*OsLEA22* and *OsLEA9* were strongly upregulated in T_CS (Table 2). LEA are extremely hydrophilic proteins that protect cellular membranes and proteins from desiccation injury (Bray, 1993; Cuming, 1999). Notably, LEA proteins cooperate with trehalose in this process (Goyal et al., 2005). Accordingly, two trehalose‐6‐phosphate synthase (*OsTPS*) genes were upregulated in T_CS (Table 2). Next, *OsUSP36* was >5‐fold upregulated in T_CS. USP36 is a member of universal stress proteins (USP) that is induced by cold stress (Arabia et al., 2021). The precise mechanism by which USPs bring about stress tolerance varies, but *OsUSP36* could be a target of DREB1a as its promoter includes a *DRE* element. Further, two genes encoding chaperon proteins dnaJ were also markedly upregulated in T_CS, possibly providing protective function to cellular components (Table 2). Finally, rice *Early Response to Dehydration7* (*ERD7*) was strongly upregulated in T_CS. Arabidopsis ERD7 accumulates in response to multiple abiotic stresses (Cheng et al., 2013; Rasmussen et al., 2013), and a recent study reported its association with phospholipids and membranes and the susceptibility of *erd7* mutant to cold stress (Barajas‐Lopez et al., 2021).

### 3.10 Secondary metabolites

Several genes involved in secondary metabolite production were markedly upregulated in T_CS that include genes encoding geranylgeranyl pyrophosphate synthase (GGPS), terpene synthases (TPS), phenylpropanoid (lignin) pathway, and polyamine oxidase (PAO). *OsGGPS*, *OsTPS3*, and *OsTPS23* were upregulated four‐ to fivefold in T_CS, suggesting a role for terpenoids in DREB1a‐mediated stress tolerance. Jung et al. (2021) found induction of *OsTPS3* as part of the mechanism associated with improved drought tolerance in rice. Phenylpropanoid pathway genes *OsCCR* and *OsC4H* that encode cinnamoyl‐CoA reductase and cinnamate 4‐hydroxylase, respectively, were upregulated >6‐fold in T_CS (Table 2). These genes are involved in lignin biosynthesis, and lignin is a well‐known cell wall biopolymer that imparts wall strength (Vanholme et al., 2010). Rice genome contains seven polyamine oxidase (*OsPAO*) genes that are involved in back‐conversion of polyamines toward maintaining polyamine homeostasis during stress (Ono et al., 2012; Yu et al., 2019). *OsPAO4* and *OsPAO6* were upregulated three‐ to fourfold in T_CS (Table 2). Sagor et al. (2021) found that *OsPAO4* and *OsPAO6* are rapidly induced by abiotic stresses including cold, salinity, drought, and wounding. The catalytic activity of POA leads to H_2_O_2_ production that presumably functions as a signal molecule in the defense pathways (Angelini et al., 2010; Gupta et al., 2016).

### 3.11 Environment sensing and signal transduction

Protein phosphorylation, cell communication, signal transduction, and chemical response were among GO biological processes associated with upregulated genes in T_CS, and plasma membrane, cell periphery, and intrinsic component of plasma membrane were associated with GO cellular component (Table 1). Accordingly, a number of receptor‐like kinases (RLK) were markedly upregulated to four‐ to sevenfold and contained *DRE* elements. For example, wall‐associated receptor like kinase (*OsDEES1*; Os09g056160) was upregulated 7.5‐fold, Ser/Thr receptor‐like kinase (*OsRLCK253a*; Os08g0374600) was upregulated 6.7‐fold, putative leucine‐rich repeat (LRR) receptor‐like protein kinase At2g19210 (Os05g0524500) was upregulated 4.9‐fold, serine/threonine‐protein kinase NAK (Os04g0563900) was upregulated 4.5‐fold, and LRR receptor‐like serine/threonine‐protein kinase GSO2 (Os01g0162200) was upregulated 4‐fold. Additionally, Ca++ sensing proteins such as calmodulin (*OsCAM1*; Os03g0319300) and calcium‐dependent protein kinase (*CDPF*; Os08g0540400) were upregulated to 2.0‐ and 4.1‐fold, respectively. Over‐representation of these genes in T_CS suggests early sensing of the environmental stress and efficient signal transduction in *RD29a:DREB1a* transgenic rice plants.

### 3.12 Rice DREB genes

Rice DREB1 gene family (*OsDREB1*) is upregulated in the early phase (2–6 h) of the cold stress at 4–10°C (DasGupta et al., 2020; Dubouzet et al., 2003; Yun et al., 2010). To check if *OsDREB* genes play a synergistic effect in stress tolerance in *RD29a:DREB1a* transgenic plants, expression of *OsDREB1* and *OsDREB2* genes was compared between genotypes and treatment (Figure S4). Although *OsDREB1* gene family has been described to express similarly in response to cold stress (Mao & Chen, 2012), we observed variable pattern among *OsDREB1* gene family. *OsDREB1C* (Os06g0127100) and *OsDREB1G* (Os02g0677300) were upregulated either by cold treatment in non‐transgenic line (N_CS) or in the transgenic line irrespective of the treatment (T_RT and T_CS), suggesting a positive effect of Arabidopsis DREB1a on these genes. Other *OsDREB1* genes such as *OsDREB1A*, *OsDREB1B*, and *OsDREB1H* were cold induced in both genotypes, and *OsDREB2* paralogs generally remained unaltered between the two genotypes or by the treatment (Figure S4). *OsDREB1C* and *OsDREB1G* are significantly upregulated by drought, cold, and flooding (Mittal et al., 2012; Mohanty, 2021; Wang et al., 2011) and found to be highly expressed in drought‐ or cold‐tolerant rice lines (Chawade et al., 2013; Jung et al., 2017). A recent study demonstrated a direct role of *OsDREB1G* in cold tolerance by developing *OsDREB1G* over‐expressor rice (*Japonica* cv. Dongjin) that showed tolerance to cold but not to drought or salinity (Moon et al., 2019). Search of *DRE* elements in the promoter regions of *OsDREB1C* and *OsDREB1G* showed that *OsDREB1G* contains three copies of DRE motif ACCGAC. Arabidopsis DREB1a binds more efficiently to this motif than OsDREB1A, providing an explanation for higher induction of *OsDREB1G* in *DREB1a* transgenic rice as compared with the non‐transgenic controls.

### 3.13 Downregulated genes

GO analysis and a survey of the top downregulated genes in T_CS in comparison with N_CS showed that genes associated with response to heat such as heat‐shock proteins and heat‐shock factors were significantly downregulated in T_CS and photosynthesis‐related genes such as protochlorophyllide reductase, chlorophyll a/b‐binding proteins, and photosystem II were downregulated in T_CS (Figure S3a,b). One of the downregulated (4.5‐fold) heat‐shock factors is *OsHsfB2b* (Os08g0546800), a Class B heat‐shock factor, found to negatively regulate drought and salt stress in rice (Xiang et al., 2013). Downregulation of heat response and photosynthesis genes was not found in T_RT and N_RT or N_CS and N_RT comparisons (data not shown). Therefore, the set of downregulated genes in T_CS possibly reflects DREB1a‐mediated reprogramming of the cellular response by not only activating defense but also curbing processes that may interfere in the dehydration stress response.

### 3.14 RNA‐seq data validation

To confirm RNA‐seq data, quantitative PCR analysis was conducted on the upregulated DEGs which contained *DRE* elements with following criteria: (i) DEGs co‐expressed under abiotic stress conditions, (ii) TF genes co‐expressed under abiotic stress conditions, and (iii) possible direct targets of DREB1a but not necessarily co‐expressed. The first category included three genes *OsRBOH* (Os12g0541300), *OsJAZ5* (Os03g0402800), and *OsJAZ11* (Os04g0395800). The second category involved four transcription factors *OsDREB1a* (Os09g0522200), *OsDERF5* (Os027g0764700), *ONAC059* (Os01g0862800), *OsbHLH148*, and *OsWRKY76* (Os09g0417600), and the third category included *OsFLS2* (Os04g0618700), *MAPKKK17_18* (Os05g0545300), OsCAM1 (Os03g0319300). This analysis found a good correlation between RNA‐seq and qPCR (*R* ^2^ = .85) and generally validated RNA‐seq data (Figure S5).

## 4 DISCUSSION

Multigene stacking via Cre‐*lox* recombination had previously generated heritable expression of a stacked locus that contained Arabidopsis *DREB1a* regulated by *RD29a* promoter along with four marker genes (Pathak & Srivastava, 2020). Arabidopsis DREB1a is a well‐known master regulator of abiotic stress tolerance (Shinozaki & Yamaguchi‐Shinozaki, 2000), and *RD29a* is abundantly induced within 3–6 h of imposing cold, osmotic, drought or salt stress (see Arabidopsis eFP Browser; Winter et al., 2007).

Abiotic stresses such as drought, cold, and salinity induce common gene networks including transcription factors, protein kinases, receptor‐like kinases, hormone signaling, and genes associated with the production of osmoprotectants that help plant survive during the stress (DasGupta et al., 2020; Todaka et al., 2015). However, most cultivated rice varieties require further improvement in abiotic stress tolerance (Menguer et al., 2017), and *DREB1a* overexpression is an attractive approach for enhancing abiotic stress tolerance. Oh et al. (2005) expressed *DREB1a* using the maize ubiquitin‐1 promoter in rice (cv. Nakdong) and observed improved tolerance to cold, salinity, and drought. In microarray analysis, Oh et al. found induction of a set of genes including *Dip1* or *Dehydrin1*, *Lip5* or *WSI724*, *Bowman Birk trypsin inhibitor2*, *Receptor kinase LRR repeats*, and *Protein Phosphatase 2C*. These genes are also upregulated >2‐fold in our transgenic rice upon cold treatment (T_CS) (Table S4). Studying chrysanthemum expressing *RD29a:DREB1a*, Ma et al. (2010) found upregulation of dehydrins and alcohol dehydrogenase (*ADH*). Similarly, we found upregulation of two *ADH* genes in T_CS (Table S4). In 10‐day‐old rice seedlings (cv. Nipponbare) expressing Arabidopsis or rice *DREB1a* under *35S* promoter, Ito et al. (2006) found upregulation of *α‐amylase*, *Lip5*, *β‐1,3‐glucanase*, and *protease inhibitor* genes, among others. These genes are also upregulated in our transgenic plants under cold stress. Notably, *β‐1,3‐glucanase* was upregulated 7.5‐fold and *α‐amylase* to 2.4‐fold in T_CS (Table S4). These genes probably function in the stress tolerance in transgenic rice. Finally, Latha et al. (2019) performed microarray on flag leaf of transgenic (*RD29a:DREB1a*) *indica* rice subjected to drought for 14 days at the booting stage. They found upregulation of a large set of genes associated with chloroplast structure and function and suggested enhanced photosynthesis during stress as part of the mechanism for transgenic rice to withstand the stress. In our dataset, however, photosynthesis and chlorophyll functions were downregulated in T_CS (Figure S3a,b). This difference could be based on the developmental stage of plants as Latha et al. used flag leaves, while we used 10‐day‐old seedlings. Clearly, their plants were much more photosynthesis active in the greenhouse compared with our seedlings grown on artificial media in relatively lower light intensity.

Each study, including ours, looked at a snapshot of transcriptome in a single tissue type and found similar genes such as dehydrins, protein kinases, and osmoprotectants in DREB1a‐expressing plants. Overall, our transgenic plants upon cold treatment showed upregulation of well‐known cold, drought, or salt stress genes described previously by others. However, a few differences are noteworthy. First, we observed enrichment of genes associated with JA signaling that is now recognized as an important part of abiotic stress regulation (Kazan, 2015; Santino et al., 2013). Both JA biosynthesis genes and repressors of JA signaling (JAZ) were upregulated in T_CS (Table 2). This is consistent with the reports that JA level increases during cold stress (Hu et al., 2013), and overexpression of *OsJAZ9* that attenuates JA signaling leads to salinity tolerance in rice (Wu et al., 2015). Second, we observed enrichment of *WRKY* TF in T_CS. Only one study, so far, has described differential expression of *WRKY* in *RD29a:DREB1a* transgenic plants (*Salvia miltiorrhiza*) (Wei et al., 2016). *OsWRKY76* that is associated with cold stress tolerance (Yokotani et al., 2013) was among the highly upregulated *WRKYs* in our study (Table 2). Next, we also observed a set of *OsERF* upregulated in T_CS, four of which are upregulated >4‐fold (Table 2). Of these, *OsERF67*, *OsERF27*, *OsERF109a*, and *OsERF109b* were reported to be highly upregulated by cold stress in 7‐day‐old seedlings of *indica* rice cv. Pusa Basmati and in a cold‐tolerant *japonica* rice cv. Jumli Marshi (Chawade et al., 2013; Mittal et al., 2012). In addition to cold stress, *OsERFs* have also been found to be associated with submergence and drought stress (Wang et al., 2011; Wu & Yang, 2020).

Finally, we observed downregulation of heat response and photosynthesis genes in T_CS. Heat stress involves a distinct mechanism based on the induction of heat‐shock proteins and heat‐shock factors, suppression of which could positively regulate response to dehydration stress. Accordingly, Xiang et al. (2013) found that overexpression of *OsHsfB2b* in rice conferred heat tolerance but also rendered them more sensitive to drought and salt stress. Downregulation of photosynthesis genes was also a unique observation in our study. Multiple chlorophyll a/b binding protein genes among other photosynthesis genes were significantly downregulated in T_CS (Figure S6), indicating suppression of primary metabolism to possibly divert energy to stress tolerance pathways.

In conclusion, expression of *RD29a:DREB1a* from a stacked locus consisting of genes expressed by constitutive and inducible promoters was effective in enhancing salinity and drought tolerance in rice. Global transcriptomic analysis of the transgenic plants showed a complex interaction of transcription factors and hormone signaling toward reprograming the plant's metabolic and growth pattern and enhancing the production of protective proteins and secondary metabolites (Figure 5). Rapid induction of the stress response through early sensing of the stress, abundant expression of the stress regulon as well as its timely attenuation, and suppression of antagonistic pathways such as heat stress is likely the basis of improved stress tolerance in transgenic rice expressing *RD29a:DREB1a* gene.

## CONFLICT OF INTEREST

Authors declare no conflict of interest.

## AUTHOR CONTRIBUTIONS

BP and YVB carried out salinity experiments, YVB and FB designed and performed drought experiments, BP carried out RNA‐seq and related bioinformatics analysis and qPCR, CM carried out DRE search, YVB and FB analyzed phenotypic data, and VS conceptualized the project, analyzed overall data, and wrote the paper.

## Supporting information


**Figure S1:** Salinity stress assay. Representative non‐transgenic (N) and RD29a:DREB1a (T) seedlings exposed to 0, 100 or 150 mM of NaCl for 12 (a‐b) or 15 (c‐d) days. Note the seedling length in (a‐b) and leaf necrosis (arrows) in (c‐d) under salt stress.

**Figure S2:** Water withholding assay. Representative plants of *RD29a:DREB1a* transgenic lines (T) and non‐transgenic (N) lines subjected to dorught stress by water withholding. Photographs were taken at 10% SWC.

**Figure S3:** Gene ontology (GO) terms, biological processes (BP), cellular components (CC), and molecular functions (MF) enriched in **(a)** up‐regulated genes and **(b)** down‐regulated genes in cold‐shocked *RD29a:DREB1a* transgenic lines in comparison to cold‐shocked non‐transgenic lines (FDR cutoff 0.01). Number of genes is given and corresponds to the size of the dots. Data analyzed on iDEP.94 (http://bioinformatics.sdstate.edu/idep94/).

**Figure S4:** Relative expression of *OsDREB1* and *OsDREB2* regulon in *RD29a:DREB1a* transgenic (T) and non‐transgenic (N) under cold‐shock (CS) or room temperature (RT) control conditions. Normalized counts of mapped reads were used to generate the heatmap.

**Figure S5:** Validation of RNA‐seq data by quantitative Reverse Transcriptase PCR (qRT‐PCR). (a) Log2 fold change in the expression of selected genes in RD29a:DREB1a transgenic lines at room temperature (T_RT) or upon cold‐shock (T_CS) as determined by RNAseq (white bars) or qRT‐PCR (pink bars). Fold change in gene expression was calculated relative to non‐transgenic controls in the respective treatment (RT or CS). (b) Correlation of RNA‐seq and qRT‐PCR data (log2 fold change).

**Figure S6:** Gene set enrichment analysis (GSEA) showing down‐regulated pathways in cold‐shocked *RD29a:DREB1a* transgenic line in comparison to cold‐shocked non‐transgenic lines. The associated GO biological processes (BP), molecular functions (MF), and cellular components (CC) are indicated. Pathway significance cut off FDR = 0.01, number of top pathways = 30. Number of genes is given and corresponds to the size of the dots. Data analyzed using iDEP.94 (http://bioinformatics.sdstate.edu/idep94/) using GAGE method (Luo et al. BMC Bioinformatics 10, 161, 2009, 10.1186/1471‐2105‐10‐161).

**Table S1:** Primers used in this study

**Table S2:** RNA‐seq raw data and quality

**Table S3:** Transcript read counts in multigene stacked transgenic line (T) upon cold stress (CS) or room temperature (RT) exposure

**Table S4:** Other genes induced by DREB1a in rice
