# Supplementary material for: Phenotypic and transcriptomic analysis reveals early stress responses in transgenic rice expressing Arabidopsis DREB1a
Source: Plant Direct. 2022 Oct 19;6(10):e456. doi: 10.1002/pld3.456 (PMC9579989; doi:10.1002/pld3.456)
Supplement: Supplementary file 1 — Figure S1: Salinity stress assay. Representative non‐transgenic (N) and RD29a:DREB1a (T) seedlings exposed to 0, 100 or 150 mM of NaCl for 12 (a‐b) or 15 (c‐d) days. Note the seedling length in (a‐b) and leaf necrosis (arrows) in (c‐d) under salt stress. [file PLD3-6-e456-s004.pptx]

## Slide 1
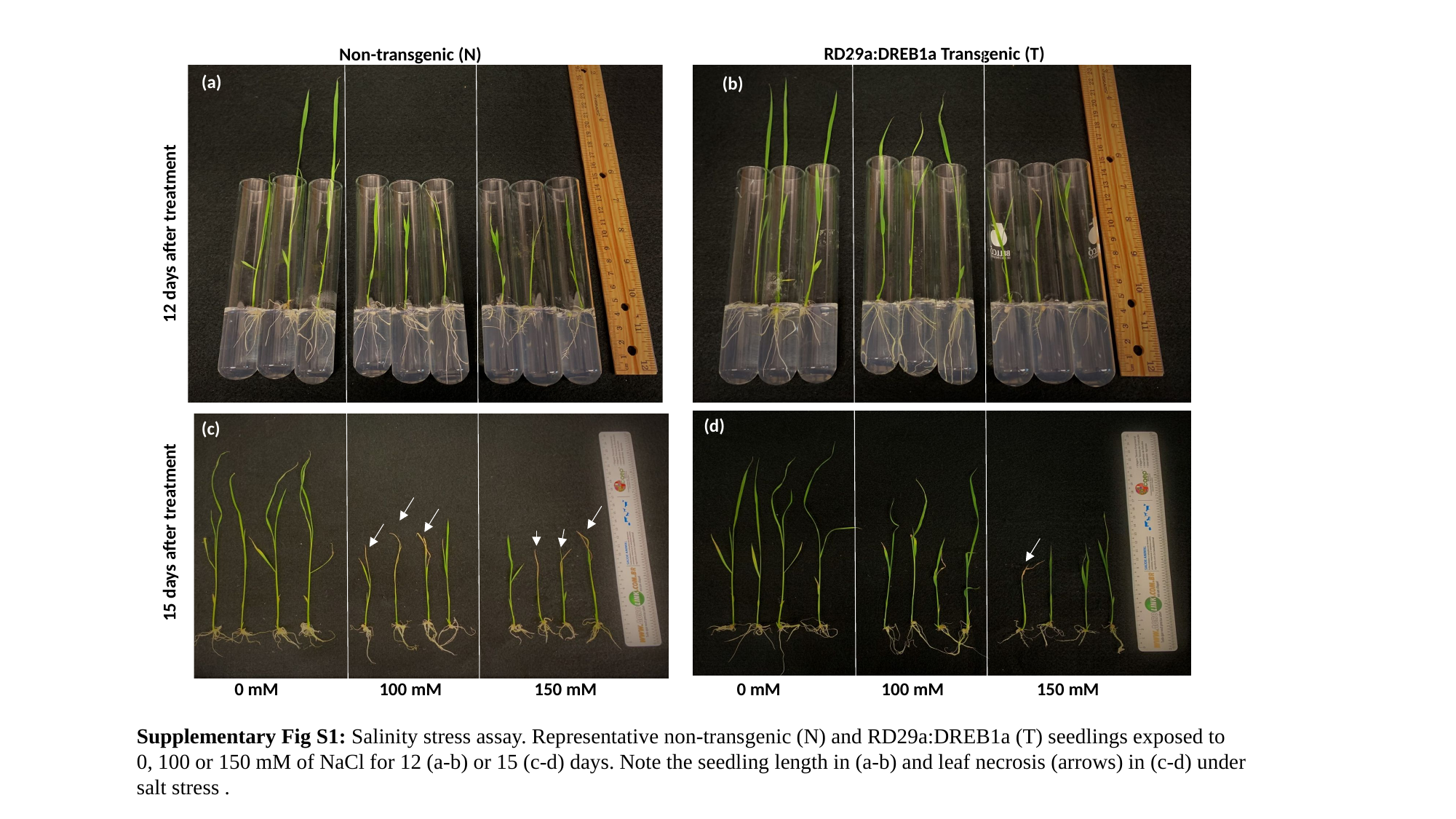

RD29a:DREB1a Transgenic (T)
Non-transgenic (N)
(a)
(b)
12 days after treatment
(d)
(c)
15 days after treatment
0 mM
100 mM
150 mM
0 mM
100 mM
150 mM
Supplementary Fig S1: Salinity stress assay. Representative non-transgenic (N) and RD29a:DREB1a (T) seedlings exposed to 0, 100 or 150 mM of NaCl for 12 (a-b) or 15 (c-d) days. Note the seedling length in (a-b) and leaf necrosis (arrows) in (c-d) under salt stress .
