# Supplementary material for: Phenotypic and transcriptomic analysis reveals early stress responses in transgenic rice expressing Arabidopsis DREB1a
Source: Plant Direct. 2022 Oct 19;6(10):e456. doi: 10.1002/pld3.456 (PMC9579989; doi:10.1002/pld3.456)
Supplement: Supplementary file 2 — Figure S2: Water withholding assay. Representative plants of RD29a:DREB1a transgenic lines (T) and non‐transgenic (N) lines subjected to dorught stress by water withholding. Photographs were taken at 10% SWC. [file PLD3-6-e456-s009.pptx]

## Slide 1
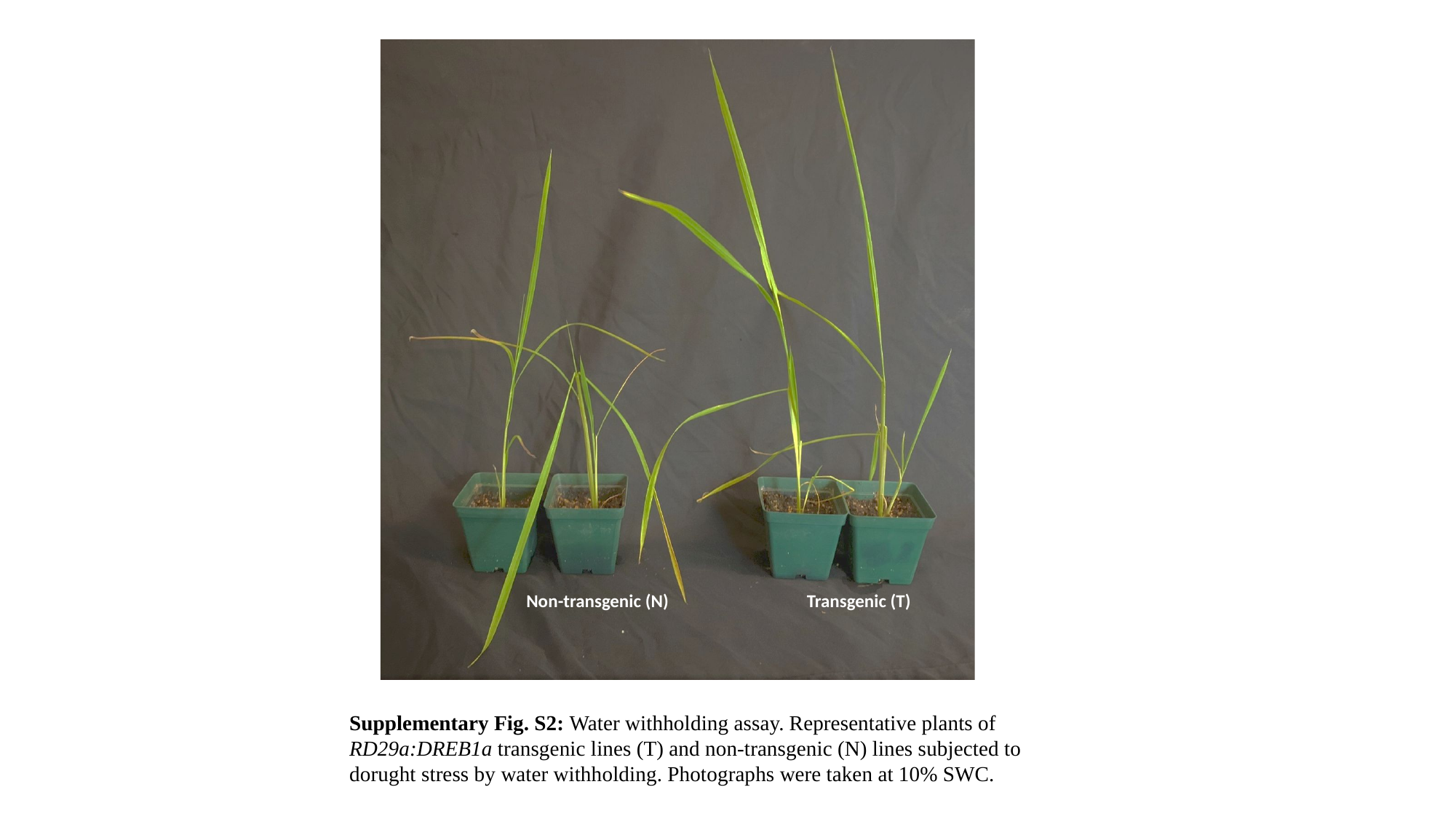

Non-transgenic (N)
Transgenic (T)
Supplementary Fig. S2: Water withholding assay. Representative plants of RD29a:DREB1a transgenic lines (T) and non-transgenic (N) lines subjected to dorught stress by water withholding. Photographs were taken at 10% SWC.
