# Supplementary material for: Phenotypic and transcriptomic analysis reveals early stress responses in transgenic rice expressing Arabidopsis DREB1a
Source: Plant Direct. 2022 Oct 19;6(10):e456. doi: 10.1002/pld3.456 (PMC9579989; doi:10.1002/pld3.456)
Supplement: Supplementary file 3 — Figure S3: Gene ontology (GO) terms, biological processes (BP), cellular components (CC), and molecular functions (MF) enriched in (a) up‐regulated genes and (b) down‐regulated genes in cold‐shocked RD29a:DREB1a transgenic lines in comparison to cold‐shocked non‐transgenic lines (FDR cutoff 0.01). Number of genes is given and corresponds to the size of the dots. Data analyzed on iDEP.94 (http://bioinformatics.sdstate.edu/idep94/). [file PLD3-6-e456-s002.pdf]

(a) Up-regulated GO terms

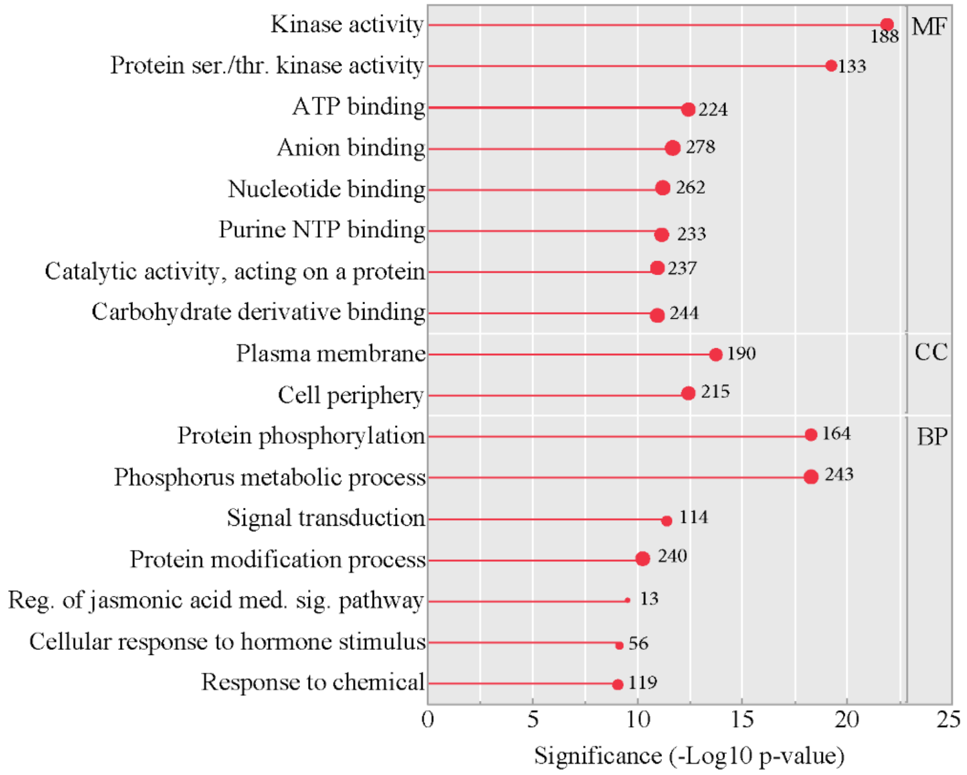

(b) Down-regulated GO terms

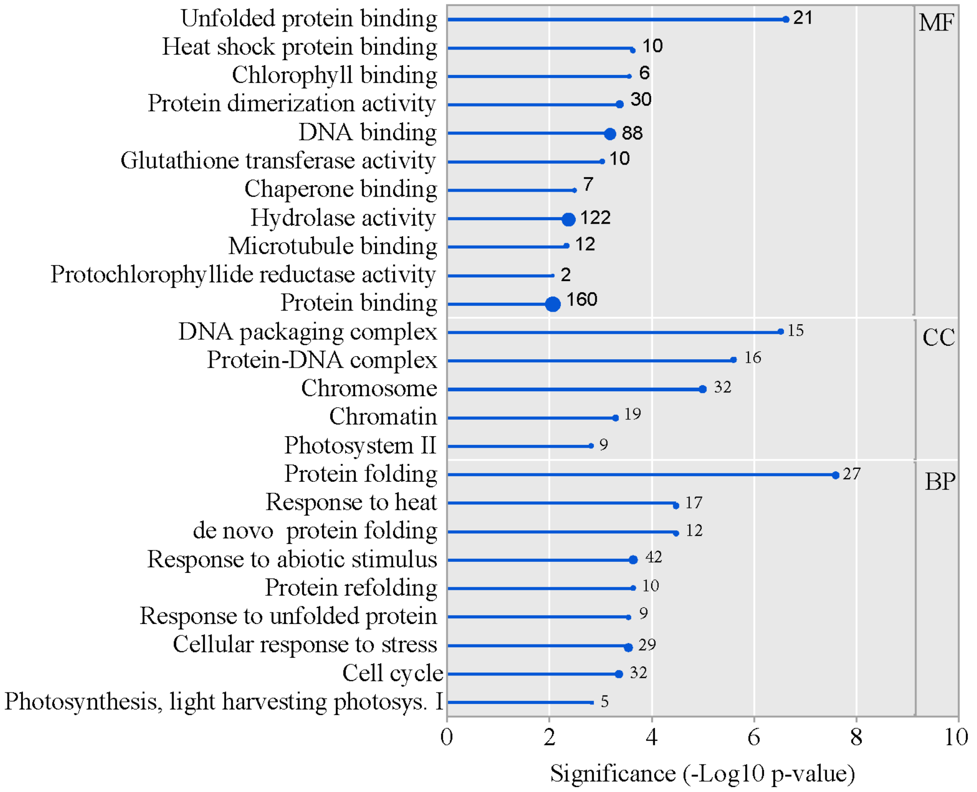

Supplementary Fig. S3: Gene ontology (GO) terms, biological processes (BP), cellular components (CC), and molecular functions (MF) enriched in **(a)** up-regulated genes and **(b)** down-regulated genes in cold-shocked *RD29a:DREB1a* transgenic lines in comparison to cold-shocked non-transgenic lines (FDR cutoff 0.01). Number of genes is given and corresponds to the size of the dots. Data analyzed on iDEP.94 (http://bioinformatics.sdstate.edu/idep94/).
