# Supplementary material for: Phenotypic and transcriptomic analysis reveals early stress responses in transgenic rice expressing Arabidopsis DREB1a
Source: Plant Direct. 2022 Oct 19;6(10):e456. doi: 10.1002/pld3.456 (PMC9579989; doi:10.1002/pld3.456)
Supplement: Supplementary file 4 — Figure S4: Relative expression of OsDREB1 and OsDREB2 regulon in RD29a:DREB1a transgenic (T) and non‐transgenic (N) under cold‐shock (CS) or room temperature (RT) control conditions. Normalized counts of mapped reads were used to generate the heatmap. [file PLD3-6-e456-s008.docx]

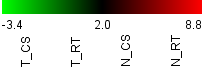


**
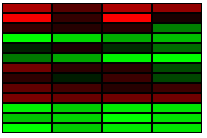
**

**OsDREB1A**

**OsDREB1B**

**OsDREB1C**

**OsDREB1D**

**OsDREB1E**

**OsDREB1F**

**OsDREB1G**

**OsDREB1H**

**OsDREB2A**

**OsDREB2A**

**OsDREB2A**

**OsDREB2A**

**OsDREB2E**

**Supplementary Fig. S4:** Relative expression of *OsDREB1* and *OsDREB2* regulon in *RD29a:DREB1a* transgenic (T) and non-transgenic (N) under cold-shock (CS) or room temperature (RT) control conditions. Normalized counts of mapped reads were used to generate the heatmap.
