# Supplementary material for: Phenotypic and transcriptomic analysis reveals early stress responses in transgenic rice expressing Arabidopsis DREB1a
Source: Plant Direct. 2022 Oct 19;6(10):e456. doi: 10.1002/pld3.456 (PMC9579989; doi:10.1002/pld3.456)
Supplement: Supplementary file 5 — Figure S5: Validation of RNA‐seq data by quantitative Reverse Transcriptase PCR (qRT‐PCR). (a) Log2 fold change in the expression of selected genes in RD29a:DREB1a transgenic lines at room temperature (T_RT) or upon cold‐shock (T_CS) as determined by RNAseq (white bars) or qRT‐PCR (pink bars). Fold change in gene expression was calculated relative to non‐transgenic controls in the respective treatment (RT or CS). (b) Correlation of RNA‐seq and qRT‐PCR data (log2 fold change). [file PLD3-6-e456-s005.docx]

**
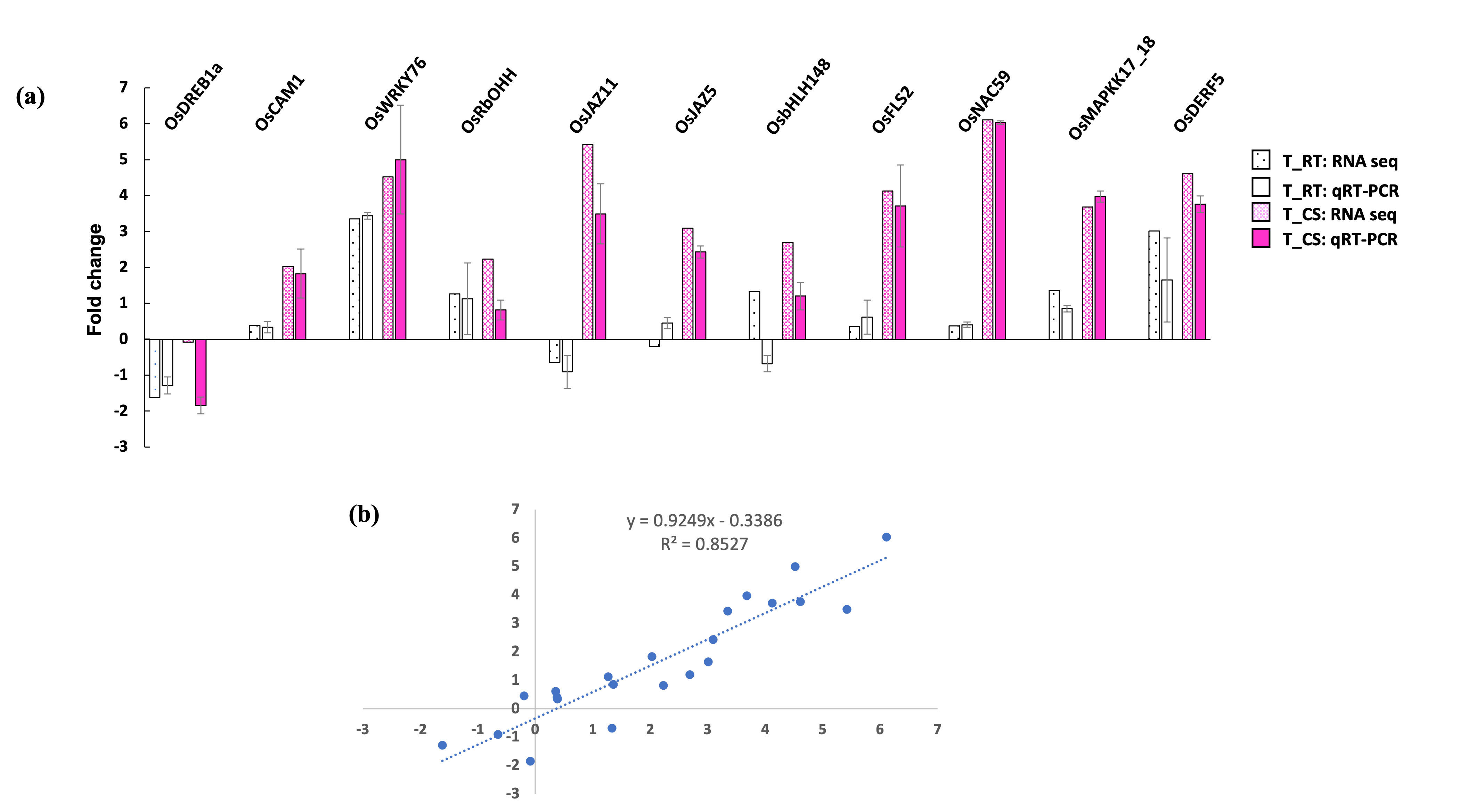
**

**Supplementary Fig. S5:** Validation of RNA-seq data by quantitative Reverse Transcriptase PCR (qRT-PCR). (**a**) Log2 fold change in the expression of selected genes in *RD29a:DREB1a* transgenic lines at room temperature (T_RT) or upon cold-shock (T_CS) as determined by RNAseq (white bars) or qRT-PCR (pink bars). Fold change in gene expression was calculated relative to non-transgenic controls in the respective treatment (RT or CS). (**b**) Correlation of RNA-seq and qRT-PCR data (log2 fold change).
