# Supplementary material for: Phenotypic and transcriptomic analysis reveals early stress responses in transgenic rice expressing Arabidopsis DREB1a
Source: Plant Direct. 2022 Oct 19;6(10):e456. doi: 10.1002/pld3.456 (PMC9579989; doi:10.1002/pld3.456)
Supplement: Supplementary file 6 — Figure S6: Gene set enrichment analysis (GSEA) showing down‐regulated pathways in cold‐shocked RD29a:DREB1a transgenic line in comparison to cold‐shocked non‐transgenic lines. The associated GO biological processes (BP), molecular functions (MF), and cellular components (CC) are indicated. Pathway significance cut off FDR = 0.01, number of top pathways = 30. Number of genes is given and corresponds to the size of the dots. Data analyzed using iDEP.94 (http://bioinformatics.sdstate.edu/idep94/) using GAGE method (Luo et al. BMC Bioinformatics 10, 161, 2009, 10.1186/1471‐2105‐10‐161). [file PLD3-6-e456-s007.pdf]

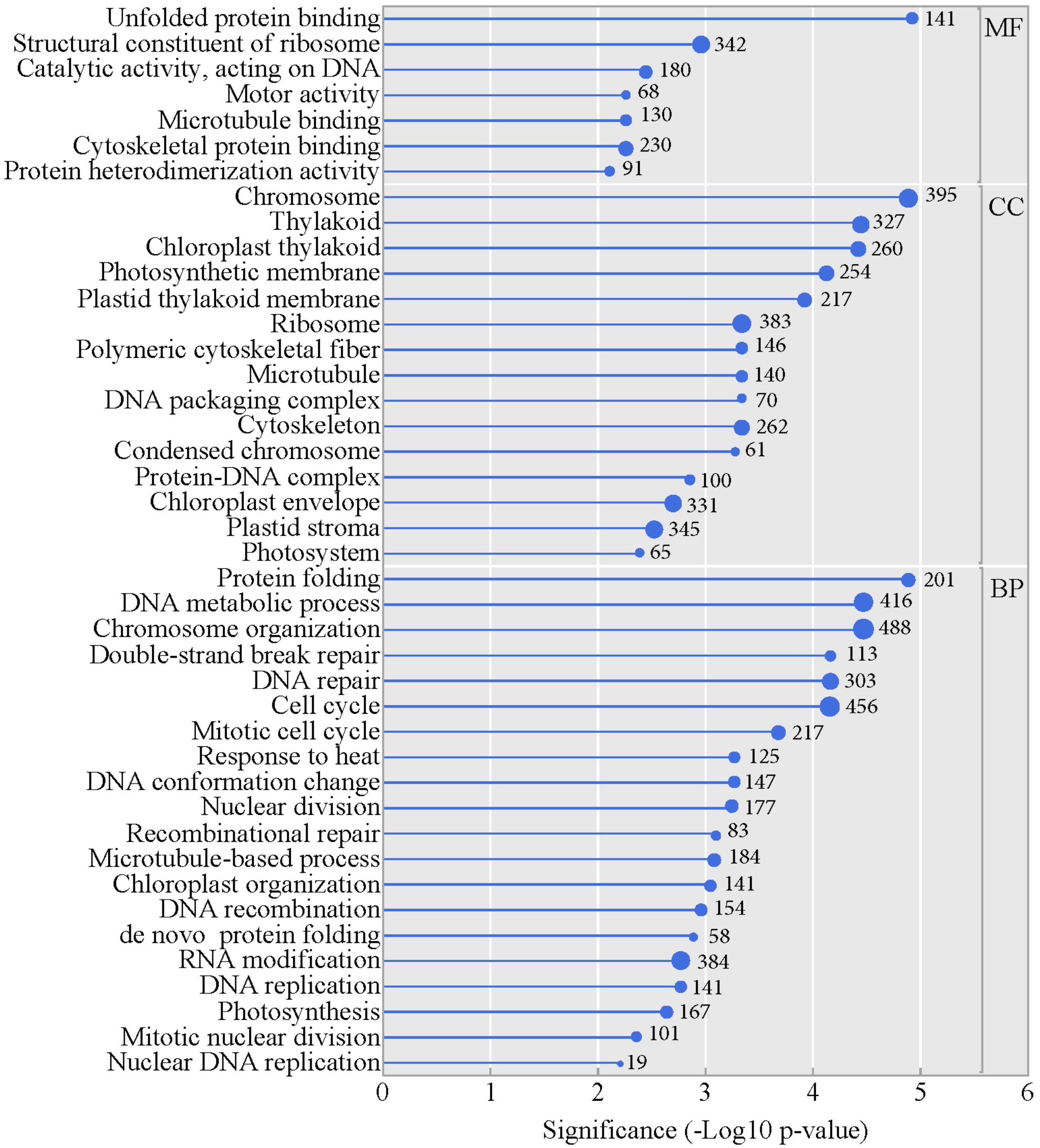

Supplementary Fig. S6: Gene set enrichment analysis (GSEA) showing down-regulated pathways in cold-shocked *RD29a:DREB1a* transgenic line in comparison to cold-shocked non-transgenic lines. The associated GO biological processes (BP), molecular functions (MF), and cellular components (CC) are indicated. Pathway significance cut off FDR = 0.01, number of top pathways= 30. Number of genes is given and corresponds to the size of the dots. Data analyzed using iDEP.94 (http://bioinformatics.sdstate.edu/idep94/) using GAGE method (Luo et al. BMC Bioinformatics 10, 161, 2009, https://doi.org/10.1186/1471-2105-10-161).
