# Supplementary material for: Phenotypic and transcriptomic analysis reveals early stress responses in transgenic rice expressing Arabidopsis DREB1a
Source: Plant Direct. 2022 Oct 19;6(10):e456. doi: 10.1002/pld3.456 (PMC9579989; doi:10.1002/pld3.456)
Supplement: Supplementary file 9 — Table S3: Transcript read counts in multigene stacked transgenic line (T) upon cold stress (CS) or room temperature (RT) exposure [file PLD3-6-e456-s010.docx]

| **Gene construct in pNS64^1^** | **Gene** | **NCBI accession no.** | **Read Counts^2^** | | **Fold Change upon CS** |
| --- | --- | --- | --- | --- | --- |
|  |  |  | **T_CS** | **T_RT** |  |
| ZmUbi:NPTII | *NPTII* | KT184682.1 | 45,353 | 25,704 | 1.7 |
| 35Sppdk:GFP | *GFP* | U55762.1 | 42,362 | 34,775 | 1.2 |
| 35S:GUS | *GUS* | S94464 | 35,158 | 18,875 | 1.8 |
| RD29a:DREB1a | *DREB1A* | NM_118680.2 | 149 | 3 | 49.6 |
| GmHSP17.5E:pporRFP | *pporRFP* | DQ206380.1 | 6 | - | ~ |

^1^pNS64 harboring 4 gene constructs was used for Cre-*lox* mediated site-specific integration into rice genome. The resulting transgenic lines were used in this study

^2^Mapped read counts of cold-stressed (CS) or room temperature control (RT) transgenic lines (T)
