# Supplementary material for: Phenotypic and transcriptomic analysis reveals early stress responses in transgenic rice expressing Arabidopsis DREB1a
Source: Plant Direct. 2022 Oct 19;6(10):e456. doi: 10.1002/pld3.456 (PMC9579989; doi:10.1002/pld3.456)
Supplement: Supplementary file 10 — Table S4: Other genes induced by DREB1a in rice [file PLD3-6-e456-s006.pdf]

Supplementary Table 4: Other genes induced by DREB1a in rice

**Gene**	**Gene ID**	**Log_2 FC[1]**	**No. of DRE[2]**
Dip1/Dehydrin1	Os02g0669100	2.4	8
Lip5/Water Stress Inducible 724	Os03g0655400	2.1	3
Bowman-Birk type Trypsin Inhibitor	Os01g0127600	4.5	5
Receptor kinase LRR repeats	Os05g0486100	2.4	4
Protein Phosphatase 2C	Os11g0242200	6.8	0
Alcohol dehydrogenase 1	Os11g0210300	2.2	1
Alcohol dehydrogenase 2	Os11g0210500	3.3	0
β-1,3-glucanase	Os01g0947000	7.5	1
α-amylase	Os01g0357400	2.4	1

[1]Fold change (log2) in cold-treated transgenic line (T_CS) compared to the cold-treted non-transgenic controls (N_CS)
[2]Number of DRE core motif (A/GCCGAC) in 2 kb sequence upstream of the start site
